# Supplementary material for: MetaRibo-Seq measures translation in microbiomes
Source: Nat Commun. 2020 Jun 29;11:3268. doi: 10.1038/s41467-020-17081-z (PMC7324362; doi:10.1038/s41467-020-17081-z)
Supplement: Supplementary file 10 — Supplementary Data 7 [file 41467_2020_17081_MOESM10_ESM.zip › File2/Confidence_High_Taxonomy/82618_out.krona.html]

Javascript must be enabled to view this page.

members
magnitude
magnitudeUnassigned
count
unassigned
taxon
rank

82618\_out

151

151
2
superkingdom

1
1239
phylum

1
909932
class

order
1843488
1

1
909930
family

1
33024
genus

33025
species

SRS098644\_contig\_number\_24681
1

150
976
phylum

150
class
200643

150
171549
order

family
815
150

150
genus
816

species
818

SRS056273\_contig\_number\_13310SRS146813\_contig\_number\_26712SRS893378\_contig\_number\_18976
3

141

SRS011134\_contig\_number\_46868SRS012849\_contig\_number\_21929SRS012969\_contig\_number\_1976SRS013098\_contig\_number\_23542SRS013521\_contig\_number\_9202SRS013965\_contig\_number\_42072SRS014235\_contig\_number\_46292SRS014313\_contig\_number\_1661SRS014412\_contig\_number\_12907SRS014459\_contig\_number\_30636SRS014613\_contig\_number\_12279SRS014683\_contig\_number\_509SRS015694\_contig\_number\_22260SRS015960\_contig\_number\_1051SRS016018\_contig\_number\_contig-100\_1584.96771SRS016203\_contig\_number\_903SRS017103\_contig\_number\_2537SRS017191\_contig\_number\_13389SRS017433\_contig\_number\_2661SRS017821\_contig\_number\_contig-100\_304.129590SRS018049\_contig\_number\_contig-100\_1756.20105SRS018133\_contig\_number\_11607SRS018313\_contig\_number\_4531SRS018427\_contig\_number\_contig-100\_1070.123149SRS018541\_contig\_number\_12724SRS018623\_contig\_number\_17706SRS018936\_contig\_number\_contig-100\_36.87926SRS019030\_contig\_number\_243SRS019161\_contig\_number\_25227SRS019178\_contig\_number\_4493SRS019267\_contig\_number\_6372SRS019397\_contig\_number\_2948SRS020394\_contig\_number\_5966SRS021219\_contig\_number\_5588SRS022071\_contig\_number\_37536SRS023583\_contig\_number\_240SRS023715\_contig\_number\_27454SRS023914\_contig\_number\_23743SRS024492\_contig\_number\_15045SRS042966\_contig\_number\_19581SRS043001\_contig\_number\_5362SRS043667\_contig\_number\_2386SRS043701\_contig\_number\_14948SRS043768\_contig\_number\_29651SRS043841\_contig\_number\_11277SRS044535\_contig\_number\_18791SRS045826\_contig\_number\_11889SRS046068\_contig\_number\_682SRS047044\_contig\_number\_11429SRS047433\_contig\_number\_17297SRS048060\_contig\_number\_9716SRS048164\_contig\_number\_contig-100\_1107.115223SRS049773\_contig\_number\_2423SRS049995\_contig\_number\_24832SRS050752\_contig\_number\_29607SRS050925\_contig\_number\_14098SRS050941\_contig\_number\_20052SRS050998\_contig\_number\_5867SRS052078\_contig\_number\_1408SRS053398\_contig\_number\_10843SRS053573\_contig\_number\_829SRS054352\_contig\_number\_15943SRS054905\_contig\_number\_26095SRS058723\_contig\_number\_2821SRS062427\_contig\_number\_7942SRS062654\_contig\_number\_18376SRS063370\_contig\_number\_11941SRS063518\_contig\_number\_36802SRS064757\_contig\_number\_15714SRS065397\_contig\_number\_13563SRS074964\_contig\_number\_908SRS075821\_contig\_number\_22328SRS075984\_contig\_number\_19055SRS076756\_contig\_number\_2978SRS077024\_contig\_number\_4819SRS077086\_contig\_number\_11713SRS077294\_contig\_number\_15716SRS077335\_contig\_number\_3262SRS077392\_contig\_number\_23322SRS077502\_contig\_number\_8647SRS077589\_contig\_number\_1041SRS077849\_contig\_number\_12051SRS078176\_contig\_number\_contig-100\_60.148605SRS078419\_contig\_number\_25137SRS078665\_contig\_number\_8050SRS098571\_contig\_number\_18517SRS098827\_contig\_number\_4674SRS101376\_contig\_number\_contig-100\_250.163761SRS103987\_contig\_number\_26466SRS1041031\_contig\_number\_30233SRS1041039\_contig\_number\_24485SRS1041090\_contig\_number\_6037SRS1041092\_contig\_number\_10900SRS1041118\_contig\_number\_6509SRS1041134\_contig\_number\_10440SRS1041139\_contig\_number\_632SRS1041141\_contig\_number\_12872SRS104327\_contig\_number\_1571SRS104400\_contig\_number\_8884SRS104975\_contig\_number\_23561SRS105082\_contig\_number\_18515SRS1054716\_contig\_number\_14503SRS1055076\_contig\_number\_9156SRS142542\_contig\_number\_7445SRS142599\_contig\_number\_2881SRS142781\_contig\_number\_1351SRS142890\_contig\_number\_7678SRS142923\_contig\_number\_23943SRS143181\_contig\_number\_2620SRS143342\_contig\_number\_9861SRS143523\_contig\_number\_21710SRS143598\_contig\_number\_5402SRS143722\_contig\_number\_6920SRS143876\_contig\_number\_25762SRS143895\_contig\_number\_20085SRS144183\_contig\_number\_21820SRS144362\_contig\_number\_7850SRS144603\_contig\_number\_contig-100\_9.109832SRS147039\_contig\_number\_contig-100\_312.40027SRS147346\_contig\_number\_9327SRS147425\_contig\_number\_8192SRS147557\_contig\_number\_33078SRS147614\_contig\_number\_3446SRS147977\_contig\_number\_7348SRS148091\_contig\_number\_10397SRS148253\_contig\_number\_12331SRS148319\_contig\_number\_13930SRS148424\_contig\_number\_2176SRS148817\_contig\_number\_8728SRS149181\_contig\_number\_27060SRS149244\_contig\_number\_4680SRS149784\_contig\_number\_22665SRS149879\_contig\_number\_3278SRS150029\_contig\_number\_4842SRS892882\_contig\_number\_15091SRS893230\_contig\_number\_12691SRS893279\_contig\_number\_13284SRS893285\_contig\_number\_168SRS893295\_contig\_number\_10895SRS893327\_contig\_number\_5489SRS971276\_contig\_number\_9010
47678
species


SRS104311\_contig\_number\_29761SRS148196\_contig\_number\_42538SRS893382\_contig\_number\_1764
3
46506
species


SRS045244\_contig\_number\_2795
1
674529
species

2

SRS011084\_contig\_number\_28760SRS021484\_contig\_number\_26164
1263037
species
